# A retrospective study on the efficacy and safety of anlotinib combined with limb salvage therapy for osteosarcoma after chemotherapy failure

**DOI:** 10.1097/MD.0000000000048632

**Published:** 2026-05-22

**Authors:** Yang Sun, Daoyang Fan, Tao Jin, Zhuoyu Li, Weifeng Liu

**Affiliations:** a Department of Orthopedic Oncology, Beijing Jishuitan Hospital, Capital Medical University, Beijing, China.

**Keywords:** anlotinib, chemotherapy, osteosarcoma, tyrosine kinase inhibitors

## Abstract

This study aimed to evaluate the efficacy and safety of anlotinib combined with limb salvage therapy in patients with osteosarcoma after chemotherapy failure. A retrospective study was conducted on 46 eligible osteosarcoma patients who failed chemotherapy, admitted to our hospital from October 2021 to December 2022. They were divided into 2 groups: 28 patients in the anlotinib combined with limb salvage surgery group (combined group) and 18 patients in the limb salvage surgery alone group (surgery group). The primary endpoint was progression-free survival (PFS); secondary endpoints included objective response rate, disease control rate, overall survival (OS), and adverse events (AEs). Therapeutic efficacy was evaluated by response evaluation criteria in solid tumors 1.1, and AEs were graded by common terminology criteria for adverse events v4.0. The median PFS in the combined group was 4.8 months (95% confidence interval: 3.5–7.1 months), significantly longer than that in the surgery group (2.8 months, *P* < .05). The objective response rate and disease control rate in the combined group were 39.29% and 67.86%, respectively, which were numerically higher than those in the surgery group (16.67% and 38.89%). Common AEs in the combined group included anorexia, leukopenia, and elevated transaminases, with significantly higher incidence than in the surgery group (*P* < .05); hypothyroidism was more common in the surgery group (*P* < .05). Anlotinib combined with limb salvage therapy shows favorable efficacy in prolonging PFS and improving disease control in chemotherapy-refractory osteosarcoma patients, with manageable adverse reactions. It may be a viable option for such patients.

## 1. Introduction

Osteosarcoma (OS) is the most common primary malignant bone tumor in children and young adults, with a median age of onset of 20 years and an annual incidence of approximately 3 per million.^[1,2]^ The main factors affecting prognosis include the location and size of the tumor, the presence and location of metastasis, response to chemotherapy, type of surgery, and so on. Since the 1970s, chemotherapy has been used in the treatment of osteosarcoma, and the 5-year survival rate of patients has increased from <20% to nearly 70%.^[3–5]^ Currently, preoperative (“neoadjuvant”) systemic polychemotherapy followed by local surgical therapy and then postoperative (“adjuvant”) chemotherapy has become the standard treatment approach for osteosarcoma.^[6]^ Recommended chemotherapy regimens include doxorubicin, high-dose methotrexate, cisplatin, and ifosfamide, although not all patients respond favorably to these treatments. Poor efficacy of first-line and second-line chemotherapy drugs can often hinder complete tumor resection and limb reconstruction during subsequent surgery, leading patients to face the possibility of amputation or even life-threatening consequences.^[7]^

Small molecule tyrosine kinase inhibitors (TKIs) have demonstrated efficacy in adjuvant treatment for various malignant tumors.^[8,9]^ Anlotinib, a novel multitargeted TKI drug recently approved by the China Food and Drug Administration, effectively inhibits vascular endothelial growth factor receptor 2 (VEGFR2), platelet-derived growth factor receptor β (PDGFRβ), and fibroblast growth factor receptor 1 (FGFR1), thereby halting tumor growth and metastasis. Given the critical role of angiogenesis in the occurrence and progression of many tumors, particularly in osteosarcoma (OS), targeted anti-angiogenic agents have been widely applied in oncological therapy in recent years. Among these, TKIs represent a major class of therapeutic agents. For instance, erlotinib, targeting the epidermal growth factor receptor (EGFR), is primarily used for the treatment of non-small cell lung cancer and pancreatic cancer. Sorafenib, targeting VEGFR, is indicated for advanced renal cell carcinoma and hepatocellular carcinoma. Dasatinib, targeting PDGFR, is used for the treatment of chronic myeloid leukemia and acute lymphoblastic leukemia.^[10,11]^ Additionally, multi-targeted inhibitors such as anlotinib have demonstrated efficacy in suppressing tumor angiogenesis, blocking nutrient supply to tumors, and thereby inhibiting tumor growth. Moreover, anlotinib exerts antitumor effects by interfering with multiple biological functions of tumor cells. Current therapeutic strategies for OS include radical surgery, chemotherapeutic agents, and combination therapies. While these approaches have improved patient survival to some extent, the prognosis for patients with recurrent or metastatic OS remains poor, with survival rates remaining around 20%. Tumor progression is influenced by a complex interplay of factors, including aberrant gene regulation, dysregulated metabolic enzyme activity, and genetic factors. Notably, abnormal activation of protein tyrosine kinases and metabolic dysregulation lead to alterations in downstream signaling pathways associated with tumorigenesis. TKIs are primarily employed in the treatment of advanced malignancies, such as chemorefractory or recurrent OS, and often yield satisfactory initial therapeutic responses, significantly improving disease-free survival. However, prolonged use of TKIs is associated with the development of drug resistance and adverse effects. Reduction or discontinuation of TKIs due to treatment-related toxicities can significantly impact patient prognosis, drawing increasing attention to the side effects of these agents. The mechanisms underlying tumor resistance to TKIs remain incompletely understood and warrant further investigation.

Anlotinib, a novel multi-target TKI, exerts its antitumor effects by targeting multiple sites, including VEGFR, FGFR, PDGFR, and c-kit, thereby inhibiting tumor angiogenesis and suppressing tumor growth. Preclinical studies have shown that anlotinib targets VEGFR2 and MET, inhibiting the growth and metastasis of osteosarcoma. Studies have indicated that anlotinib demonstrates positive antitumor activity in inoperable or metastatic primary bone malignancies. In a phase II clinical trial reported by Li et al, 29 patients with recurrent or metastatic osteosarcoma were treated with anlotinib, achieving a partial response (PR) in 2 patients, a disease control rate (DCR) of 75.86%, and a median progression-free survival (PFS) of 4.8 months, indicating some clinical efficacy. A phase IIB randomized trial has shown that anlotinib can significantly prolong PFS in patients with advanced sarcoma. Li et al observed VEGF amplification in 23% of osteosarcoma patients, with a significant increase in its primary receptor VEGFR protein, suggesting that the VEGFR signaling pathway is responsive to TKIs. In an IB/II phase clinical study of sarcoma, although anlotinib in combination with irinotecan and vincristine did not achieve the expected results, it significantly reduced the risk of hypertension in patients. Anlotinib has demonstrated remarkable clinical efficacy in various solid tumors, including non-small cell lung cancer and soft tissue sarcoma. However, its effects on osteosarcoma remain under-studied. This study aims to analyze the efficacy and safety of anlotinib in combination with chemotherapy for osteosarcoma and evaluate the significance of anlotinib-based chemotherapy as a neoadjuvant intervention after first-line chemotherapy failure in osteosarcoma patients.

## 2. Patients and methods

### 2.1. Research design

This single-center retrospective study was conducted at Beijing Jishuitan Hospital in accordance with the Declaration of Helsinki and was approved by the Ethics Committee of Beijing Jishuitan Hospital (approval No. 2023-9-18). Eligible patients were identified between October 2021 and December 2022. Written informed consent for treatment and use of clinical data was obtained from all participants or their legal guardians.

### 2.2. Patients

Patients were eligible for inclusion if they met all of the following criteria: Histologically confirmed osteosarcoma; age ranging from 15 to 70 years; confirmed ineligibility for radiotherapy or surgical intervention; Eastern Cooperative Oncology Group Performance Status score of 0 or 1; no prior history of treatment with other targeted agents; assessable disease according to the response evaluation criteria in solid tumors (RECIST) version 1.1; all enrolled patients had received either the methotrexate, doxorubicin, cisplatin regimen (methotrexate 12 g/m^2^ on days 1 and 8, doxorubicin 75 mg/m^2^ by 72-hour continuous infusion, cisplatin 120 mg/m^2^ intra-arterial, cycles were repeated every 3 weeks, 2–3 preoperative cycles were standard) or the methotrexate, doxorubicin, cisplatin plus ifosfamide regimen (methotrexate 12 g/m^2^ on days 1 and 8, doxorubicin 75 mg/m^2^ by 72-hour continuous infusion, cisplatin 120 mg/m^2^ intra-arterial, Ifosfamide 2–3 g/m^2^/day × 3–5 days, cycles were administered every 3 weeks, 2 preoperative cycles were typically given) for 2 cycles without satisfactory response; therefore, patients whose best outcome after 2 cycles was stable disease or progressive disease were deemed to have experienced chemotherapy failure; and acceptable hematological parameters, hepatic function, and renal function, along with availability of complete pathological, imaging, and clinical records. This was a descriptive analysis, and the follow-up period was extended until December 30, 2023. The medical history, previous treatments, clinical outcomes, and demographic characteristics of all enrolled patients were comprehensively evaluated.

Patients were excluded if they had any of the following conditions: Patients with tumors breaking through the knee joint cavity and surrounding ligaments; patients involving important surrounding blood vessels and nerves; patients with tumor progression or distant metastasis after preoperative neoadjuvant chemotherapy; uncontrolled blood pressure, active myocardial ischemia, history of arterial infarction, and cardiac insufficiency; patients who have undergone major surgery within 4 weeks; patients with venous thrombosis within 6 months; and patients with infectious complications.

### 2.3. Anlotinib treatment

The treatment doses for adults and adolescents are 12 and 10 mg, respectively, administered orally once daily before breakfast for 2 consecutive weeks, followed by a 1-week drug holiday. This 3-week treatment cycle is repeated. If patients miss the first dose, they are instructed to skip the missed dose if <12 hours remain until the next scheduled dose.

### 2.4. Limb salvage surgery

Based on imaging examinations conducted before and after neoadjuvant chemotherapy, the precise location, size, and relationship with the surrounding tissue structures of the tumor were determined. The surgical boundary necessary for tumor resection was delineated, encompassing the extent of normal soft tissue resection and osteotomy length. During surgery, standard disinfection and draping procedures were followed, with meticulous care taken to identify and safeguard vital nerves and blood vessels. Upon exposure of the lesion, the tumor along with its surrounding tissue, extending 3 to 5 cm from the tumor edge, was completely excised to ensure adequate margins. After complete resection of the tumor segment lesion, prostheses were implanted to reconstruct the absent bone and joint structures. If necessary, ligaments and muscle attachment points were also reconstructed. Hemostasis was ensured, drainage tubes were placed, and the wound was closed with sutures.

In surgical management, the approach should be tailored to the patient’s specific condition, such as the extent of tissue defect or vascular and nerve injury. Techniques may include microsurgical repair for small vessels and nerves, bone grafting, or bone transport techniques for bone defects. In rehabilitation, early passive exercises can prevent joint stiffness and promote circulation, while mid-term active exercises enhance muscle strength and joint mobility. Late-stage rehabilitation focuses on weight-bearing and proprioceptive training to restore limb function and coordination, supplemented by physical and occupational therapy. Postoperative care involves regular wound dressing and monitoring for healing and infection. Pain management follows medical advice, with analgesics and relaxation techniques. Psychological support addresses emotional changes, and personalized rehabilitation plans guide patients through training, emphasizing safety precautions. Regular follow-up via outpatient visits, phone, or online communication ensures timely adjustment of the treatment plan.

### 2.5. Postoperative follow-up program

The surgery was performed at week 9, 2 weeks after the completion of the 2 cycles of chemotherapy before surgery. Twenty-eight cases of limb salvage surgery and drug combined treatment, there were 18 cases treated with limb salvage surgery alone. Follow-up visits were performed once a month for 6 months and once every 3 months for 2 years. During the follow-up, pulmonary computed tomography (CT) and X-ray pictures of the lesion site were reviewed every 3 months. Follow-up was conducted every 6 months. Time of recurrence, metastasis, and death was recorded.

### 2.6. Outcomes

The primary endpoint was PFS, defined as the time from the date of limb-salvage surgery to documented disease progression or death from any cause, whichever occurred first. Secondary endpoints included objective response rate (ORR), defined as the proportion of evaluable patients with complete response or PR, DCR, defined as the proportion of patients with complete response, PR, or stable disease, and overall survival (OS), defined as the time from the date of limb-salvage surgery to death from any cause. Tumor response was assessed according to RECIST version 1.1.

### 2.7. Assessment of safety

Adverse events (AEs) were assessed in the safety-evaluable population, which included patients with complete AE monitoring records during the predefined observation period from treatment initiation to 1 month after treatment completion or discontinuation. AEs were graded according to common terminology criteria for adverse events version 4.0. The frequency, severity, and clinical relevance of AEs were analyzed descriptively.

### 2.8. Statistical analysis

All data analyses were performed using SPSS 20.0, following the intention-to-treat principle. For clinical baseline data and AE data, direct counting was adopted. Changes in intragroup baseline values for continuous variables were analyzed using the *t*-test, and continuous variables were expressed as mean ± standard deviation, median (range), or median (interquartile range [IQR]). Categorical variables were presented as frequencies and percentages, with changes in intragroup baseline values analyzed using the chi-square (χ^2^) test or Fisher exact test where appropriate. PFS and overall survival (OS) were estimated using the Kaplan–Meier method and expressed as median (95% confidence interval). All tests were two-tailed, with the significance level set at α = 0.05.

## 3. Results

### 3.1. Patient characteristics

**Figure 1.**
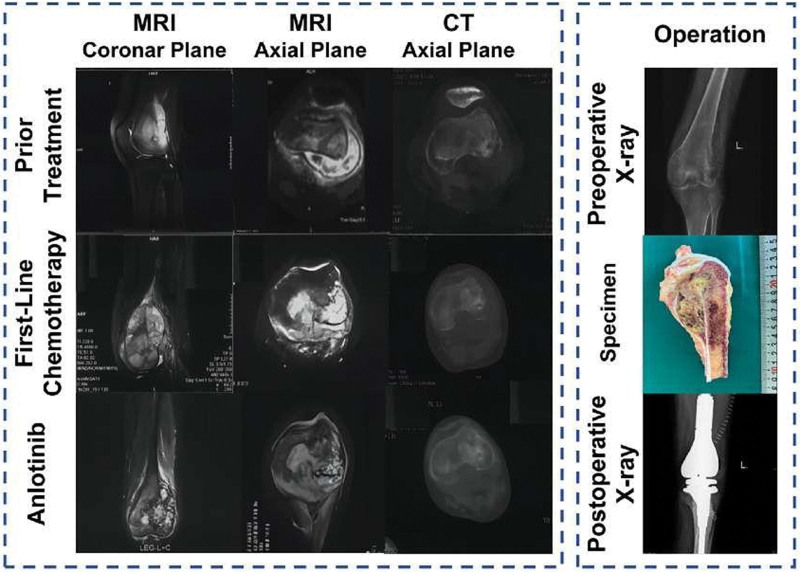
A typical case of combined treatment of anlotinib and limb salvage surgery. Initial MRI and CT scans in the coronal and axial planes reveal a large osteosarcoma in the distal femur prior to any treatment; post-first-line chemotherapy MRI shows the tumor with stable disease, as evidenced by minimal changes in size on comparison with pretreatment imaging; MRI and CT after anlotinib treatment demonstrate a partial response with noticeable reduction in tumor size; intraoperative X-ray and postoperative specimen images illustrate the limb salvage surgery, highlighting complete tumor resection and successful prosthetic reconstruction. Postoperative X-ray confirms the stability of the reconstructed limb. CT = computed tomography, MRI = magnetic resonance imaging.

A retrospective analysis was conducted on patients diagnosed with osteosarcoma in our hospital. A total of 46 eligible patients were enrolled in our hospital from October 2021 to 2022, among whom 28 received anlotinib combined with limb-salvage surgery (anlotinib combination therapy group) and 18 received only limb-salvage surgery (surgical treatment group). Clinical data of all patients, such as gender, age, and previous treatment history, were collected retrospectively. In the anlotinib combination therapy group, the median age of patients was 28.0 years (IQR: 18.0–36.0), with 71.43% being male and 28.57% being female. In the surgical treatment group, the median age was 26.0 years (IQR: 17.0–34.0), including 55.6% males and 44.4% females. In addition, in the anlotinib combination therapy group, 19 patients (64.29%) and 7 patients (25.00%) had stage IVA and stage IVB diseases, respectively; in the surgical treatment group, 13 patients (72.22%) and 3 patients (16.67%) had stage IVA and stage IVB diseases, respectively. All patients had received chemotherapy prior to enrollment. Figure 1 shows just a typical case of combined treatment of anlotinib and limb salvage surgery. The demographic and baseline characteristics of the patients are shown in Table 1.

**Table 1:** Demographic and baseline characteristics of the patients.

Variables	Anlotinib combination therapy group (n = 28)	Surgical treatment group (n = 18)
Age, yrs		
Median (IQR)	28.0 (18.0–36.0)	26.0 (17.0–34.0)
Gender		
Male, n (%)	20 (71.43)	10 (55.6)
Female, n (%)	8 (28.57)	8 (44.4)
ECOG performance status score, n (%)		
0	9 (32.14)	5 (27.78)
1 and above	12 (42.86)	10 (55.56)
2 and above	7 (25.00)	3 (16.67)
Clinical stage, n (%)		
IIB	1 (3.57)	1 (5.56)
III	2 (7.14)	1 (5.56)
IVA	19 (64.29)	13 (72.22)
IVB	7 (25.00)	3 (16.67)
Previous surgery, n (%)		
Yes	28 (100)	18 (100)
Previous targeted therapy, n (%)		
Yes	4 (14.29)	3 (16.67)
Metastatic at diagnosis		
Lung metastasis	19 (67.86)	9 (50.00)
Metastasis to other sites	5 (17.86)	3 (16.67)
Adjuvant therapy, n (%)	28(100)	18 (100)
First line chemotherapy, n (%)	25 (89.29)	8 (44.44)
Second-line chemotherapy, n (%)	5 (17.86)	3 (16.67)
Third-line chemotherapy, n (%)	2 (7.14)	3 (16.67)
Unclear	1 (3.57)	2 (11.11)

ECOG = Eastern Cooperative Oncology Group, IQR = interquartile range.

### 3.2. Efficacy measures

**Figure 2.**
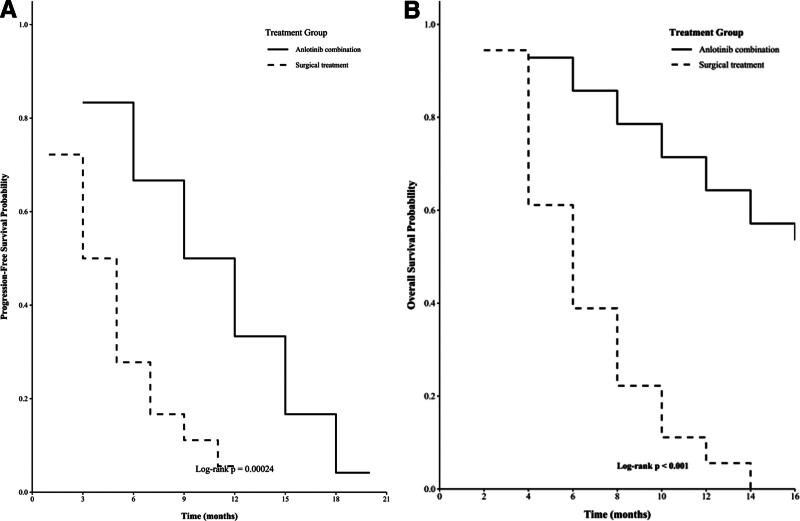
The Kaplan–Meier curve of progression-free survival (A) and overall survival (B) in the intention-to treat (ITT) population.

The survival curves in Figure 2A and B show the results of the PFS and overall survival (OS) for the patients in the 2 treatment groups. Figure 2A indicates that the median PFS of patients in the anlotinib combination treatment group (solid line) is significantly longer than that of the surgical treatment group (dashed line), which was determined by the Log-rank test (*P* = .00024, Fig. 2A). This suggests that adding anlotinib to the treatment regimen effectively delays the progression of the disease.

Further analysis of the results in Figure 2B shows that the overall survival probability of the anlotinib combination treatment group is also higher, and there is a statistically significant difference (Log-rank *P* < .001, Fig. 2B). These findings collectively indicate that the anlotinib combination treatment not only prolongs the time of disease progression but also improves the long-term survival rate of patients with osteosarcoma.

Table 2 further confirmed the overall treatment response of the 2 groups of osteosarcoma patients. In the anlotinib combination treatment group (n = 28), the ORR was 39.29%, and the DCR was 67.86%. In contrast, the ORR in the surgical treatment group (n = 18) was 16.67%, and the DCR was 38.89%. The difference in ORR between the 2 groups was statistically significant (*P* = .03, Table 2). The DCR in the anlotinib combination treatment group significantly increased (*P* = .012, Table 2). This indicates that the anlotinib combination treatment has a stronger ability to control the disease, which may contribute to the observed survival advantage.

**Table 2:** The overall treatment response of osteosarcoma.

	N	CR	PR	SD	PD	ORR (%)	DCR (%)
Anlotinib combination therapy group	28	0	11	8	9	39.29%	67.86%
Surgical treatment group	18	0	3	4	11	16.67%	38.89%
*P*						.03	.012

Response levels were evaluated based on independent radiological reviews in accordance with RECIST version 1.1.

CR = complete response, DCR = disease control rate, ORR = objective response rate, PD = progressive disease, PR = partial response, SD = stable disease.

### 3.3. Adverse reaction

AEs were analyzed in the safety-evaluable population rather than the entire cohort because complete AE monitoring data were not available for all included patients. Therefore, Table 3 summarizes AEs in 19 patients from the combined group and 8 patients from the surgery group. In the combined group, the most common AEs included anorexia, anemia, elevated transaminases, leukopenia, and thrombocytopenia. Compared with the surgery group, anorexia, leukopenia, and elevated transaminases occurred more frequently in the combined group (all *P* < .05). Most AEs were manageable with routine supportive care and monitoring (*P* < .05; Table 3).

**Table 3:** Statistical analysis of adverse reactions.

Adverse events (AEs)	Anlotinib combination therapy group (n = 19)	Surgical treatment group (n = 8)	*P*
Hypertension	1 (5.26%)	0 (0.0%)	.525
Anemia	1 (5.26%)	1 (12.5%)	.658
Leukopenia	3 (15.79%)	1 (12.5%)	.045
Thrombocytopenia	3 (15.79%)	2 (25.0%)	.432
Fatigue	1 (5.26%)	1 (12.5%)	.658
Hand-foot syndrome	0 (0.0%)	0 (0.0%)	.588
Anorexia	4 (21.05%)	0 (0.0%)	.000
Transaminase elevation	3 (15.79%)	0 (0.0%)	.047
Diarrhea	1 (5.26%)	0 (0.0%)	.525
Pneumothorax	1 (5.26%)	0 (0.0%)	.525
Hypothyroidism	1 (5.26%)	3 (37.5%)	.045

## 4. Discussion

Osteosarcoma, as the most common primary bone tumor in pediatric and adolescent populations, has long been managed with the multimodal treatment approach of “neoadjuvant chemotherapy – surgery – adjuvant chemotherapy” as the standard protocol. Thanks to this regimen, the prognosis of osteosarcoma patients has been significantly improved, with the 5-year survival rate elevated to 60%–80%. However, prominent therapeutic challenges still persist. On one hand, osteosarcoma exhibits a high degree of heterogeneity. The biological behaviors and histological characteristics of tumor cells vary dramatically among different patients, leading some patients to be inherently resistant to first-line chemotherapy drugs or to develop acquired resistance gradually during treatment. In cases of chemotherapy failure, patients not only face a higher risk of amputation but also experience a substantial increase in mortality risk. On the other hand, even after standardized treatment, the recurrence rate of osteosarcoma remains high. Approximately 30% of localized osteosarcomas and 80% of metastatic osteosarcomas will recur.^[12]^ Patients with recurrent disease have an extremely poor prognosis, and traditional treatment methods often fail to achieve satisfactory outcomes, highlighting an urgent need to explore new therapeutic strategies.

Numerous studies have unraveled the complex mechanisms underlying chemotherapy resistance in osteosarcoma. At the genetic level, the overexpression of drug resistance-related genes such as ABCB1 and ABCG2 can reduce intracellular drug concentrations through active efflux of chemotherapeutic agents, thereby inducing resistance. Within the tumor microenvironment, cellular components including cancer-associated fibroblasts and tumor-associated macrophages, as well as the remodeling of extracellular matrix, can affect drug delivery and efficacy, promoting the development of resistance. Additionally, the existence of cancer stem cells constitutes another crucial factor in chemotherapy resistance. These cells possess the capabilities of self-renewal and multi-lineage differentiation, and they are relatively insensitive to chemotherapeutic drugs, enabling them to survive chemotherapy and reinitiate tumor growth. For recurrent and metastatic osteosarcoma, traditional second-line chemotherapy regimens – such as ifosfamide combined with doxorubicin, or gemcitabine combined with docetaxel – exhibit limited efficacy, with median PFS often <4 months. This underscores the urgency and importance of developing novel therapeutic approaches.

Anlotinib, a novel oral multi-targeted TKI, has a mechanism of action that is closely linked to the pathogenesis of osteosarcoma. Tyrosine kinases play a pivotal role in intracellular signal transduction pathways, and their aberrant activation is highly prevalent in osteosarcoma. By regulating fundamental cellular processes such as proliferation, migration, and apoptosis, these kinases drive tumor initiation and progression.^[13]^ Anlotinib can specifically target these aberrantly activated tyrosine kinases, block signal transduction, thereby inhibiting the growth and survival of tumor cells. Additionally, it can cut off the nutritional supply to tumors through its anti-angiogenic effects. A substantial body of basic and clinical research provides robust evidence supporting the application of anlotinib in osteosarcoma treatment. In the classic KHOSR2 xenograft nude mouse model, Wang et al (2021) found that anlotinib combined with doxorubicin (DOX) not only significantly reduced tumor growth rate but also markedly shrank tumor volume. This result reveals the potential value of anlotinib in overcoming multidrug resistance of osteosarcoma to conventional chemotherapeutic agents, providing important experimental basis for the design of subsequent clinical combination regimens. Subsequent studies have further validated the efficacy of this combination therapy in nude mouse models derived from other osteosarcoma cell lines. For instance, in the model established with Saos-2 cells, anlotinib combined with DOX also significantly inhibited tumor growth. Immunohistochemical analysis showed that the expression of VEGF, a protein related to angiogenesis, was significantly reduced in tumor tissues of the combination group, and the positive rate of Ki-67, a cell proliferation marker, also decreased significantly, further explaining the advantages of combination therapy from a mechanistic perspective.

In this study, the RECIST 1.1 criteria were adopted for efficacy evaluation; however, these criteria have multiple limitations in assessing osteosarcoma. Firstly, due to the unique biological characteristics of osteosarcoma bone lesions, they are classified as “nonmeasurable diseases,” making it impossible to directly evaluate treatment efficacy through traditional measurements of changes in lesion size.^[14]^ Secondly, osteosarcoma exhibits high heterogeneity, with significant variations in the biological behaviors and histological features of tumor cells among different patients. Additionally, phenomena such as pseudoprogression and pseudoresponse – where temporary changes in tumor volume do not truly reflect treatment effects – render the RECIST 1.1 criteria ineffective in accurately assessing efficacy. Furthermore, these criteria primarily focus on changes in tumor size, neglecting important goals in osteosarcoma treatment such as improving patients’ quality of life and preserving limb function. Moreover, the limitations of conventional imaging techniques like CT and magnetic resonance imaging in detecting tiny lesions or early changes further compromise the accuracy of evaluation.^[15,16]^ To more precisely evaluate the efficacy of anlotinib in osteosarcoma treatment, future studies should integrate multi-dimensional assessment methods. On one hand, histopathological examinations should be incorporated – for instance, evaluating tumor necrosis rate to determine the therapeutic effect on tumor cell killing. On the other hand, functional imaging techniques such as PET-CT should be introduced to reflect tumor cell activity by monitoring changes in tumor metabolic activity. Simultaneously, clinical symptom assessments – including the degree of pain relief and recovery of limb mobility in patients – should be included to comprehensively judge treatment efficacy from multiple perspectives, thereby more fully and accurately reflecting the true efficacy of anlotinib. In terms of histopathological evaluation, studies analyzing osteosarcoma specimens resected after neoadjuvant therapy have found that tumor necrosis rate is closely related to patient prognosis, with a high tumor necrosis rate often indicating better survival outcomes. Therefore, dynamically monitoring changes in tumor necrosis rate is of great significance in evaluating the efficacy of anlotinib. In functional imaging, PET-CT, which utilizes differences in the uptake of radioactive tracers by tumor cells, can more sensitively detect changes in tumor cell metabolic activity. A study comparing PET-CT with traditional CT in osteosarcoma efficacy evaluation revealed that PET-CT can detect metabolic changes in tumors earlier after treatment and has higher accuracy in judging disease recurrence and assessing efficacy. Combining these techniques with clinical symptom assessments – such as evaluating improvements in symptoms like pain and limb dysfunction through patient-reported outcome scales – can construct a comprehensive and three-dimensional efficacy evaluation system, providing a more reliable basis for assessing the efficacy of anlotinib in osteosarcoma treatment.

The high ORR and DCR exhibited by anlotinib in osteosarcoma treatment hold extensive and far-reaching clinical significance.

Firstly, in terms of impact on individual patients, an improved ORR means that more patients experience significant tumor shrinkage, directly alleviating symptoms such as pain, compression, and other related symptoms, thereby markedly enhancing their quality of life.

Secondly, it has revolutionized the treatment strategies. Anlotinib provides a novel therapeutic option for specific cancer subtypes with limited options with conventional therapies, such as NTRK fusion-positive osteosarcoma, fully embodying the value of precision medicine. This suggests that detecting genetic status such as FGFR1 may help further identify populations with superior benefits from anlotinib, enabling more precise treatment. In terms of safety, there are variations in adverse reactions to anlotinib across different studies. A multicenter study showed that 54.76% of patients experienced grade 3 or higher AEs when treated with anlotinib for recurrent or metastatic primary malignant bone tumors, including hypertension (19.05%), hypertriglyceridemia (9.52%), and palmoplantar pustulosis (7.14%). However, other studies have reported that adverse reactions can be effectively controlled, and patient tolerance and adherence can be improved through optimized dosages, close monitoring, and management.

This study is limited by its retrospective single-arm design, which inherently carries certain drawbacks. The absence of a control group renders the study susceptible to various confounding biases. Exclusive reliance on RECIST 1.1, a criterion with limited sensitivity and specificity in osteosarcoma, introduced inherent measurement bias. Gold-standard histologic necrosis, metabolic imaging, and biochemical markers were not systematically collected; indeed, postoperative necrosis rates were documented in only 12 of 46 patients. Consequently, the reported efficacy outcomes should be viewed as hypothesis-generating and must be confirmed in prospective, controlled trials. In addition, owing to the limited availability of sequential imaging data, future prospective trials should incorporate standardized serial magnetic resonance imaging/CT with automated volumetric analysis to better characterize tumor response dynamics. Currently, clinical evidence supporting the efficacy of anlotinib in chemotherapy-refractory osteosarcoma remains limited, with most data derived from small retrospective studies or case series. These reports suggest modest activity of anlotinib in advanced osteosarcoma, but no study to date has demonstrated that anlotinib alone can overcome chemoresistance or significantly improve survival outcomes. Therefore, while our findings indicate potential disease control in a highly selected cohort, they should be interpreted with caution and seen as hypothesis-generating rather than definitive. Future prospective, multicenter, randomized trials are warranted to clarify whether anlotinib can truly alter the disease course or improve surgical outcomes in patients with chemo-refractory osteosarcoma. Additionally, as a single-center retrospective study spanning 2021 to 2022, we relied on routine pathology reports that did not uniformly quantify post-chemotherapy tumor necrosis; the corresponding glass slides are no longer available for central reevaluation. Consequently, we could not correlate necrosis rates with clinical outcomes. Prospective trials should mandate standardized pathological review to address this gap. To further clarify the role and value of anlotinib in osteosarcoma treatment, future research should focus on the following directions: Firstly, conduct large-scale, multicenter randomized controlled trials to directly compare anlotinib with traditional treatment regimens, thereby identifying its advantages and limitations. Secondly, deepen the exploration of biomarkers associated with anlotinib’s efficacy, such as NTRK fusion status and mutation profiles of specific tyrosine kinases, to achieve more precise patient stratification and enhance treatment effectiveness. Thirdly, implement long-term follow-up studies to comprehensively assess the long-term impact of anlotinib on patients overall survival (OS) and quality of life, while improving strategies for monitoring and managing adverse reactions, ensuring that patients derive maximum benefit from treatment under safe conditions.

Currently, several large-scale randomized controlled trials are underway. For example, a phase III study evaluating anlotinib combined with chemotherapy versus chemotherapy alone as first-line treatment for osteosarcoma is expected to provide higher-level evidence for the clinical application of anlotinib. In terms of biomarker exploration, with the popularization of next-generation sequencing technology, multicenter, large-sample genetic sequencing studies are being conducted to comprehensively decode the genomic landscape of osteosarcoma and identify key molecular markers related to anlotinib’s efficacy and drug resistance. Long-term follow-up studies are also being gradually improved: through the establishment of a national osteosarcoma patient database, patients receiving anlotinib treatment will be tracked over the long term, with a focus not only on survival data but also on multi-dimensional indicators such as quality of life and psychological status. This will provide more comprehensive information for the holistic evaluation of anlotinib’s clinical value.

In conclusion, as a multi-targeted TKI, anlotinib exhibits significant antitumor activity in the treatment of chemotherapy-resistant or progressive osteosarcoma. By improving the ORR and DCR, anlotinib can effectively prolong patients’ PFS, create favorable conditions for limb-salvage surgery, and thus represents a potentially important option for enhancing patients’ survival outcomes and quality of life. However, its clinical application still needs to be combined with an optimized efficacy evaluation system (such as the integration of pathological assessments, functional imaging, and clinical symptom evaluations) as well as precise biomarker detection. Meanwhile, large-scale randomized trials are urgently required to further verify its efficacy and safety. This study provides new ideas and clues for the precision treatment of osteosarcoma, lays a foundation for subsequent in-depth explorations, and is expected to promote new breakthroughs in the field of osteosarcoma treatment.

## Acknowledgments

We would like to thank the patients and his parents for participating in this work.

## Author contributions

**Conceptualization:** Yang Sun, Daoyang Fan, Tao Jin, Zhuoyu Li, Weifeng Liu.

**Data curation:** Yang Sun, Daoyang Fan, Tao Jin, Zhuoyu Li, Weifeng Liu.

**Formal analysis:** Yang Sun, Daoyang Fan, Tao Jin, Zhuoyu Li, Weifeng Liu.

**Funding acquisition:** Yang Sun, Weifeng Liu.

**Investigation:** Weifeng Liu.

**Writing – original draft:** Yang Sun, Zhuoyu Li, Weifeng Liu.

**Writing – review & editing:** Yang Sun, Weifeng Liu.
